# Development of a Novel System for Mass Spectrometric Analysis of Cancer-Associated Fucosylation in Plasma **α**_1_-Acid Glycoprotein

**DOI:** 10.1155/2013/834790

**Published:** 2013-02-13

**Authors:** Takayuki Asao, Shin Yazawa, Toyo Nishimura, Takashi Hayashi, Hideyuki Shimaoka, Abby R. Saniabadi, Hiroyuki Kuwano

**Affiliations:** ^1^Department of General Surgical Science, Gunma University Graduate School of Medicine, Maebashi 371-8511, Japan; ^2^Tokushima Research Institute, Otsuka Pharmaceutical Co., Ltd., Tokushima 771-0192, Japan; ^3^Institute of Biomedical Innovation, Otsuka Pharmaceutical Co., Ltd., Tokushima 771-0192, Japan; ^4^S-BIO Business Division, Sumitomo Bakelite Co., Ltd., Tokyo 140-0002, Japan; ^5^JIMRO Co., Ltd., Tokyo 151-0063, Japan

## Abstract

Human plasma **α**_1_-acid glycoprotein (AGP) from cancer patients and healthy volunteers was purified by sequential application of ion-exchange columns, and N-linked glycans enzymatically released from AGP were labeled and applied to a mass spectrometer. Additionally, a novel software system for use in combination with a mass spectrometer to determine N-linked glycans in AGP was developed. A database with 607 glycans including 453 different glycan structures that were theoretically predicted to be present in AGP was prepared for designing the software called AGPAS. This AGPAS was applied to determine relative abundance of each glycan in the AGP molecules based on mass spectra. It was found that the relative abundance of fucosylated glycans in tri- and tetra-antennary structures (FUCAGP) was significantly higher in cancer patients as compared with the healthy group (*P* < 0.001). Furthermore, extremely elevated levels of FUCAGP were found specifically in patients with a poor prognosis but not in patients with a good prognosis. In conclusion, the present software system allowed rapid determination of the primary structures of AGP glycans. The fucosylated glycans as novel tumor markers have clinical relevance in the diagnosis and assessment of cancer progression as well as patient prognosis.

## 1. Introduction 

The two main classes of glycosidic linkages to proteins involve either N-glycans or O-glycans through asparagine or serine and threonine, respectively. The N-linked glycans attached to the protein contain a trimannosyl pentasaccharide, Man*α*1,6[Man*α*1,3]Man*β*1,4GlcNAc*β*1,4GlcNAc ([Man]_3_[GlcNAc]_2_) as the common core structure, and are classified into three main groups, high mannose, complex and hybrid types [1]. The complex-type glycans have no mannose residues other than those in the common core structure but have antennae or branches with N-acetyllactosamine residues at the reducing termini attached to the common core structure.

*α*_1_-Acid glycoprotein (AGP, orosomucoid) possessing the complex type glycans is the major plasma glycoprotein with a molecular weight of 41–43 kDa. Further, this glycoprotein has highly branched N-linked glycans, bi-, tri-, and tetra-antennary glycans including elongated tetra-antennary glycans with repeating lactosamine structures having (sialyl) Le^X^ determinant, (NeuAc*α*2,3)Gal*β*1,4[Fuc*α*1,3]GlcNAc*β* [2]. 

Recently, we reported on AGP glycoforms in plasma samples from cancer patients with malignancies that differed in the degree of branching and the extent of fucosylation as determined by crossed affinoimmunoelectrophoresis (CAIE) with Con A lectin and *Aleuria aurantia* lectin (AAL) and anti-AGP antibody [3]. Accordingly, patients with advanced malignancies who had AGP glycoforms containing highly fucosylated branched glycans for long periods after surgery were found to have a poor prognosis, while patients without such glycoforms were expected to have a good prognosis irrespective of their clinical stages. 

However, in the past, the purification of AGP has commonly involved chromatography on metal chelate affinity gel and anti-AGP immobilized affinity gel, ion-exchange chromatography, CAIE with Con A lectin, hot phenol extraction, and sulphosalicylic acid precipitation [4–8]. Further, more recently, purification and characterization of glycans on AGP have been done with the aid of capillary electrophoresis [9, 10], but most of these methods are unsuitable for processing a large number of plasma samples in a short period of time. The CAIE method we recently introduced can process plasma samples without purification for investigating glycoforms because AGP can be identified by means of an anti-AGP antibody but still is not applicable to large numbers of samples if rapid processing is required [3]. This experience also indicated that better data on disease progression and outcome of postoperative patients with malignancies could be obtained from changes of plasma AGP glycoforms than from changes in the level of plasma AGP. Hence investigation of glycan structures associated with these changes became a major focus of interest and inspired us to develop an automated method for rapid processing of a large number of test samples. 

With the above background in mind, in this study, we developed a simple method for the purification of plasma AGP and obtained N-linked glycans labeled with a highly sensitive agent for mass spectrometry [11–13] after digestion of AGP with trypsin and PNGase F. There are several methods available for determining glycan structures of AGP based on spectra from mass spectrometry [14]. However, most of these seem to be difficult for carrying out comprehensive analysis of glycan structures without some knowledge of the intrinsic structures in the individual glycoconjugates. The main interest in this study was the glycosylation process of AGP glycans because there is a need for adequate knowledge on the glycans that are expected to be present in plasma AGP [3]. Therefore, first, we developed a simple method to purify plasma AGP and then analyzed N-glycans by means of mass spectrometry together with constructing operation software (AGPAS) to assist analysis of glycans in AGP. At the same time, we evaluated AGPAS by investigating glycan structures and their relative abundance in plasma AGP from cancer patients whose glycoforms had been identified previously [3]. 

Since it was clearly demonstrated in our previous study that no obvious difference between patients with respect to AGP levels in plasma samples and their clinicopathologic background after surgery, it must be postulated with high probability that changes in glycosylation of the AGP molecule after surgery could indeed be used as a novel parameter for monitoring and predicting the fate of tumor-bearing patients. 

## 2. Materials and Methods

### 2.1. Materials

DEAE Sepharose Fast Flow, SP Sepharose Fast Flow, HiTrap Desalting, HiTrap DEAE FF, and HiTrap SP FF were obtained from GE Healthcare (Amersham Place, UK). PNGase F was from Roche Applied Science (Indianapolis, USA). Anti-human AGP rabbit serum was obtained from DAKO (Carpinteria, CA, USA) and peroxidase-conjugated anti-human AGP was from Abcam (Cambridge, UK). Human AGP was purchased from SIGMA (St. Louis, MO, USA). Trypsin, DTT, 2,5-dihydroxybenzoic acid, and other reagents were obtained from Wako Pure Chemicals Co. (Tokyo, Japan). BlotGlyco was from Sumitomo Bakelite Co. (Tokyo, Japan). Blood samples were obtained from patients with various types of malignancies who were admitted to the Gunma University Hospital (Maebashi, Japan) along with the guideline for informed consent and approval from the Ethics Committee of Gunma University. Details of clinicopathological features of the patients for follow-up studies were described previously [3]. Blood samples were also obtained from randomly selected volunteers as a healthy control group. Each plasma sample was stored at −80°C until use. Protein was determined with a DC protein assay kit (Bio-Rad, Richmond, CA, USA) using bovine serum albumin as a standard. SDS-polyacrylamide gel electrophoresis was carried out using a 10/20 gradient gel (MultiGel II Mini, Cosmo Bio Co. Ltd., Tokyo, Japan). After electrophoresis, the gel was transferred to an Immobilon PVDF membrane (Millipore, Bedford, MA, USA) in a Trans-Blot SD cell (Bio-Rad). The membrane was stained with Coomassie Brilliant Blue for detecting protein bands. For detecting AGP molecule, the membrane was blocked with PBS containing 5% skim milk and then the membrane was incubated with peroxidase-conjugated anti-human AGP and stained with the VECTASTAIN ABC kit (Vector Laboratories, Inc., Burlingame, CA, USA) according to the manufacturer's instruction. 

### 2.2. Preparation and Purification of N-Glycans from Purified Plasma AGP

N-Glycans released from purified AGP preparation were labeled according to the protocol of the glycosylation kit (BlotGlyco) with a slight modification. A lyophilized AGP preparation purified from 500 *μ*L of human plasma was dissolved in 50 *μ*L of H_2_O, 25 *μ*L of the solution (20–200 *μ*g AGP) was mixed with 2.5 *μ*L of 1 M ammonium bicarbonate, 2.5 *μ*L of 120 mM DTT, and 25 *μ*L of sample to be analyzed, and the mixture was incubated at 60°C for 30 min. Then 5 *μ*L of 123 mM of iodoacetamide was added and the mixture was allowed to stand under dark at room temperature for 1 h followed by addition of 5 *μ*L of 400 units of trypsin and incubation at 37°C for 1 h. After heating at 90°C for 5 min, 27 units of PNGase F was added and the mixture was incubated overnight at 37°C. After heating at 90°C for 5 min, 20 *μ*L of the mixture was used for the following labeling step. The mixture containing N-glycans released enzymatically from AGP was treated with BlotGlyco kit to prepare methyl esterified and aoWR-labeled N-glycans according to the manufacturer's instruction and labeled glycans were obtained as a 50 *μ*L of the solution.

### 2.3. Sandwich-Type ELISA of Plasma AGP

The AGP levels were measured by a sandwich-type ELISA using anti-human AGP and horseradish peroxidase-conjugated anti-human AGP as described previously [3]. 

### 2.4. Mass Spectrometric Analysis

One *μ*L of the sample solution was mixed with 1 *μ*L of 2,5-dihydroxybenzoic acid (10 mg in acetonitrile/water, 1 : 1, v/v) and an aliquot was deposited on a MALDI target plate and allowed to dry. Mass spectrometric data were obtained using an AB Sciex MALDI TOF/TOF TM 5800 System (Applied Biosystems, Inc., Foster City, CA, USA). All spectra in the mass range of *m*/*z* 1,000 to 4,550 were obtained using a positive reflectron mode. Deisotopic masses for each peak were picked by Data Explorer software (Applied Biosystems) and exported as a mass peak list with each value of both the centroid mass and the corresponding area to the operation software, AGPAS described below. 

## 3. Results and Discussion

### 3.1. Purification of Plasma AGP

**Figure 1:**
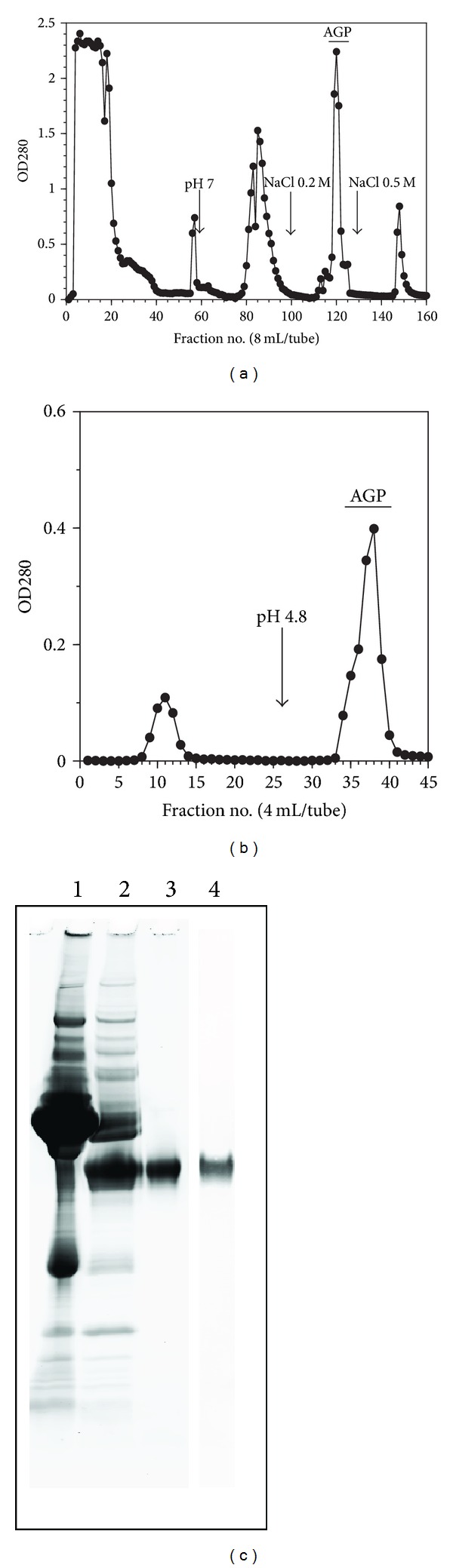
Purification of plasma AGP from pooled human plasma. (a) Ion-exchange chromatography of human plasma on a DEAE-Sepharose FF column (10 × 50 cm) equilibrated with 0.02 M citrate phosphate buffer, pH 4.0. Fifty mL of the dialyzed plasma sample was applied to the column. Fractions were assayed for protein (●) and AGP concentrations. AGP appeared only in the fractions eluted with 0.02 M citrate-phosphate buffer, pH 7.0 containing 0.2 M NaCl (**—**). (b) Ion-exchange chromatography of the eluate from DEAE-Sepharose FF column on an SP-Sepharose FF column (2 × 20 cm) equilibrated with 0.02 M citrate-phosphate buffer, pH 4.0. Ten mL of the pooled and dialyzed samples were applied to the column. Fractions were assayed for protein (●) and AGP concentration. AGP appeared in the eluate of citrate-phosphate buffer at pH 4.8 (—). (c) 10/20 SDS-polyacrylamide gel electrophoresis of purified AGP preparations. Lane 1: pooled plasma sample; lane 2: DEAE-Sepharose FF eluted fractions (0.2 M NaCl, pH 7.0); lanes 3, 4: DEAE- (0.2 M NaCl, pH 7.0) and SP- (pH 4.8) Sepharose FF eluted fractions. After electrophoresis, the gel was blotting on the membrane and each lane on the membrane was stained with Coomassie Brilliant Blue (lanes 1–3) and anti-human AGP antibody (lane 4). See the details in the Text.

Pooled human plasma (50 mL) was dialyzed against 0.02 M of citrate-phosphate buffer (pH 4.0) overnight at 4°C. After centrifugation of the dialyzed plasma at 10,000 rpm for 20 min at 4°C, the supernatant was applied to a 10 × 50 cm of DEAE-Sepharose FF column equilibrated with 0.02 M of citrate-phosphate buffer (pH 4.0) and the column was washed with the same buffer until absorbance of the eluate reached the baseline level. Then the column was washed with 0.02 M citrate-phosphate buffer (pH 7.0) containing NaCl at concentration of 0.2 M and 0.5 M, respectively. The concentrations of AGP in each fraction were monitored by means of a sandwich-ELISA method. Fractions containing AGP were eluted from the column when the column was washed with 0.02 M citrate-phosphate buffer (pH 7.0) containing 0.2 M NaCl (Figure 1(a)). All fractions containing AGP were pooled and dialyzed against 0.02 M of citrate-phosphate buffer (pH 4.0) overnight at 4°C. The dialyzed, pooled fractions were then applied to an SP-Sepharose FF column (2 × 20 cm) equilibrated with 0.02 M citrate-phosphate buffer (pH 4.0). AGP bound to the column was eluted when the column was washed with 0.02 M of citrate-phosphate buffer (pH 4.8, Figure 1(b)). This purification procedure yielded a preparation that showed a single protein band with an approximate 47 KDa on an SDS-PAGE (Figure 1(c), lane 3) and reacted with anti-human AGP antibody (Figure 1(c), lane 4). The procedure allowed over 1,000 purification fold of AGP.

For the purification of AGP in small amounts from a large number of samples, the aforementioned purification procedure was slightly modified with a sequential use of two different ion-exchange and desalting cartridge columns. A 0.5 mL sample was applied to a HiTrap Desalting, equilibrated with 0.02 M citrate-phosphate buffer (pH 4.0), and fractions at the first peak were then applied to a HiTrap DEAE FF. Fractions containing AGP were eluted with the same buffer containing 0.2 M NaCl as described above and pooled fractions were passed through two joined HiTrap Desalting columns. The eluate containing AGP was then applied to a HiTrap SP FF and AGP was eluted in the same manner as described above. The eluted fractions were lyophilized and purified AGP was then dissolved in distilled water and used for the preparation of labeled N-glycans followed by mass spectrometric analyses described above. In order to ensure a full dose of released N-glycans from AGP, levels of AGP in the purified preparations from a large number of samples were measured in advance by means of ELISA using anti-AGP antibody [3]. All the preparations for labeling and MS analysis were shown to contain adequate amounts of glycans and their labeled ones, respectively (data not shown) indicating that less than 1 *μ*L of the labeled preparations allowed to analyze most of the N-glycans expressed on the AGP molecule irrespective of their preparations both from healthy controls and patients with cancer as described below. 

The present method allowed purification of plasma AGP, and by using cartridge columns, the entire process was completed within an hour. AGP was obtained at the same purity as mentioned above. With the aid of an anti-AGP antibody-immobilized column, AGP at almost the same purity could be obtained, but it was found that some of the AGP with hypersialylated glycans were hard to react with anti-AGP antibody, passing through the column during the purification process (S. Yazawa, unpublished observations). 

### 3.2. Operation Software, AGPAS

The obtained mass spectrometric data were processed by using the assisting software we called AGPAS not only to determine N-linked glycan structures of plasma AGP automatically from the corresponding centroid masses, but also to calculate the relative abundance of individual glycans from the corresponding area relative to the total hit area. For a mass spectrometric analysis, every sample was digested with trypsin and PNGase F and then the resulting N-glycans were labeled by means of BlotGlyco. After labeling with the aoWR reagent, an exact ms of each glycan was changed as follows: MW of a labeled glycan = [an exact ms] – [H_2_O(18.0105)] + [aoWR(447.223)] + [CH_3_(14.0156) ×  *n*] + [H(1.0078)], where *n* indicates the number of N-acetylneuraminic acid residues. 

To determine the primary structures of N-glycans in AGP, the GlycoMod Tool in the ExPASY (http://web.expasy.org/glycomod/) was available, but it was not always convenient or easy to obtaine suitable results. Therefore, a database for constructing the operation software, AGPAS, was established to facilitate selection of N-glycans present in the AGP molecule with the following steps.

**Step 1**

Extract N-glycans from the GlycoMod Tool available through the ExPASy. N-Glycans having glycoform masses from 1,000 to 4,550 monoisotopic values were extracted by using the GlycoMod Tool and 4,193 glycans were selected in total.

**Step 2**

Select N-glycans of AGP. Every conceivable glycan theoretically expressed on the AGP molecule was selected based on the background knowledge. The corresponding glycans were classified as bi-, tri-, and tetra-antennary structures extending from the common core trimannosyl pentasaccharide structure attached to the protein core. These glycans are reported to consist of galactose (Gal), *N*-acetylglucosamine (GlcNAc), fucose (Fuc), and N-acetylneuraminic acid (NeuAc) residues as for [Hexose], [HexNAc], [Deoxyhexose], and [NeuAc] expressed in the GlycoMod Tool, respectively, in the case specialized for AGP glycan structures [3]. For setting a database factoring all the N-glycans expected to be present in an AGP molecule, each glycan except the common core structure was identified by using four-digit (FD) numbers indicating the number of each residue in order of Gal, GlcNAc, Fuc, and NeuAc such as FD number *abcd* for the glycan, [Gal]_a _[GlcNAc]_b _[Fuc]_c _[NeuAc]_d_ + [Man]_3_[GlcNAc]_2_. In addition, among the number of these residues, five conditional equations essential for proceeding glycosylation of AGP glycans were proposed as follows: *a* ≤ *b*, 2 ≤ *b* ≤ 7, *c* ≤ *b* − 1, *d* ≤ 4, and *a* ≥ *d* in [Gal]_*a*_ [GlcNAc]_*b*_ [Fuc]_*c*_ [NeuAc]_*d*_ (FD number *abcd*). After N-glycans corresponding to these equations were selected, the FD numbers for these N-glycans were assigned individually with their corrected MW values using software from the Visual Basic Editor in Excel (Microsoft, ver.2010, Redmond, WA, USA). Since fucosylated AGP contained only *α*-1,3-fucosylated linkages attached to GlcNAc in the tri- and tetra-antennary glycan structures [2, 3], no *α*-1,3- or *α*-1,6-fucosylated bi-antennary structures, nor *α*-1,6-fucosylated tri- or tetra-antennary structures should be present in AGP. It is likely that aberrant *α*-1,3-fucosylated biantennary glycans, and glycans having FD numbers *X21X* (*X* ≤ 2) existed but were not omitted from the database. Then, 453 glycans in total were selected.

**Step 3**

Determine the major sialylated AGP glycans. From the results of mass spectrometric analysis of randomly selected 100 samples, forty-five glycans for which the relative abundance was more than 0.1% and the number of Gal residues was more than one for sialylation were selected from N-glycans of AGP described in Step 2 as the major sialylated AGP glycans.

**Step 4**

**Figure 2:**
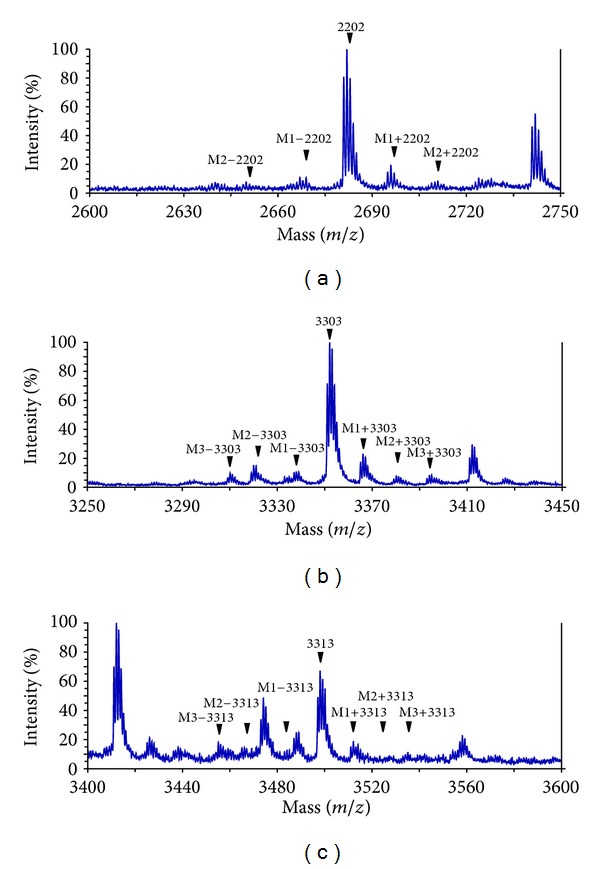
Mass spectra of methylated forms of AGP glycans derived from methyl esterification of sialylated glycans during the labeling process. Several de- (M−) and over- (M+) methylated glycans were detected around the exact labeled glycans. Purified AGP was prepared and labeled from 500 *μ*L of plasma sample and an aliquot (ca. one-fiftieth) was used for MS analysis. See the details in the text.

Create methylated glycans. During mass spectrometric analysis, a series of unidentified spectra corresponding to a group of unknown masses before and after the exact MW of sialylated glycans at ±14.0156 ×*n* *m/z* intervals (*n* = numbers of [NeuAc] residues) were observed (Figure 2). These were thought to represent methylated glycans formed from methyl esterification of the sialic acid residues attached to glycans through the labeling process undertaken in this study. Therefore, as additional glycans in the database, methylated glycans of which the numbers were theoretically calculated as lower and higher than those of [NeuAc] residues in the aforementioned major glycans with [NeuAc] residues (45 species from Step 3) were added to the database (154 glycans in total). Further, to simplify identification of these methylated glycan structures, the letter M was added to the original four-digit numbers. Therefore, methylated subpeaks such as M1−2202 indicating FD number 2202 structure with one deficient methyl residue and M4+4424 indicating FD number 4424 structure with four excess methyl residues were predicted to be present.

**Step 5**

Sum up N-glycans in the database of AGPAS. From original (452 glycans from Step 2) and additional (154 glycans, Step 4) AGP N-glycans, the numbers of glycans in the database of AGPAS approached 607 in total. Relative abundance of methylated glycans was added to the original glycans in case of making summary counts of relative abundance of individual AGP glycans. Individual centroid masses were automatically corrected based on their exact masses by using the aforementioned equation. Each corresponding area was transferred to the AGPAS for assigning labeled glycans and calculating their relative abundance. Data were then processed to identify FD numbers of glycans and to calculate their relative abundance, individually, and then a table indicating the relative abundance of bi-, tri-, and tetra-antennary glycans together with their respective fucosylated forms appeared. More than 100 peaks hit the glycans present in the database and the relative abundance of glycans totally hit was 54.63 ± 15.82% (*n* = 102).

### 3.3. Mass Spectrometric Analysis of Plasma AGP Glycans from Healthy Volunteers and Cancer Patients

**Figure 3:**
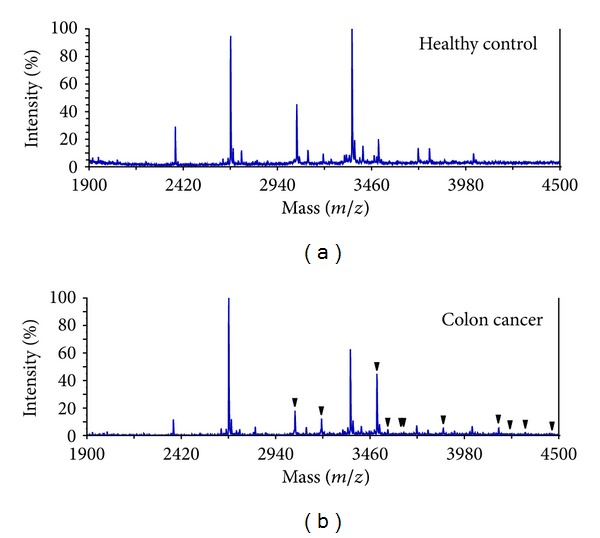
Mass spectra of AGP glycans isolated from a healthy control and a patient with colon cancer. Black triangles indicate fucosylated tri- and tetra-antennary glycans assigned. Purified AGP was prepared and labeled from 500 *μ*L of plasma samples and an each aliquot (ca. one-fiftieth) was used for MS analysis.

To evaluate the AGPAS operation software for assisting determination of glycans structures of AGP, mass spectrometric analyses of AGP glycans from healthy volunteers and patients with colorectal cancers were conducted. When mass spectra from a healthy individual and a patient with colon cancer were compared (Figure 3), signals with common masses, but different intensity, were observed in both samples. Fucosylated glycans attached to the tri-, and tetra-antennary structures seemed to increase in the cancer patient. In our previous study [3], we found that the levels of plasma AGP were not important, but the degree of branching and the extent of fucosylation in AGP glycans were very useful for predicting outcome of postoperative cancer patients. Therefore, in this study, relative abundance of each glycan both in healthy individuals (*n* = 35) and in patients with colorectal cancer (*n* = 67) was determined by using the AGPAS. Accordingly, the relative abundance of every glycan was obtained and then the individual structures were tallied simultaneously as bi-, tri-, and tetra-antennary together with their fucosylated glycans based on the second number of individual FD numbers, because it could preferentially define glycan structures from bi-, tri-, and tetra-antennary glycans (Table 1). It was clearly indicated the relative abundance of all the fucosylated tri-antennary glycans including mono- (*b* = 3,   *c* = 1 in [GlcNAc]_*b*_ [Fuc]_*c*_) and difucosylated (*b* = 3, *c* = 2 in [GlcNAc]_*b*_ [Fuc]_*c*_) structures and their total was significantly higher (*P* < 0.001) in cancer patients as compared with healthy controls even though the relative abundance of afuco-tri-antennanry glycans (*b* = 3, *c* = 0 in [GlcNAc]_*b*_ [Fuc]_*c*_) in cancer patients was significantly low (*P* < 0.001). A difference between healthy controls and cancer patients was also observed in monofucosylated (*b* = 4, *c* = 1 in [GlcNAc]_*b*_ [Fuc]_*c*_) and their afuco- (*b* = 4, *c* = 0 in [GlcNAc]_*b*_ [Fuc]_*c*_) tetra-antennary glycans (*P* < 0.001). Further, the relative abundance of fucosylated tetra-antennary glycans including mono-, di-, and tri-fucosylated (*b* = 4, *c* = 1–3 in [GlcNAc]_*b*_ [Fuc]_*c*_) glycans (*P* < 0.01) and fucosylated tri- plus tetra-antennary glycans (FUCAGP, *P* < 0.001) was significantly higher in cancer patients versus healthy controls. Therefore, it was evident that AGP glycans from cancer patients, most of whom had undergone operation and received chemotherapy periodically, were considerably fucosylated irrespective of their clinical status. It was, of particular interest, that the level of FUCAGP in cancer patients was significantly higher than the level in healthy controls but that no such a difference was present in the tri- plus tetra-antennary glycans (*b* = 3–7, *c* = 0 in [GlcNAc]_*b*_ [Fuc]_*c*_). It should be appropriate to mention here that AGP glycoforms have been investigated in patients with noncancerous diseases, and elevated plasma AGP and fucosylated AGP were associated with inflammation [2, 15–22]. 

### 3.4. Mass Spectrometric Analysis of Plasma AGP Glycans in Followed Up Cancer Patients

**Figure 4:**
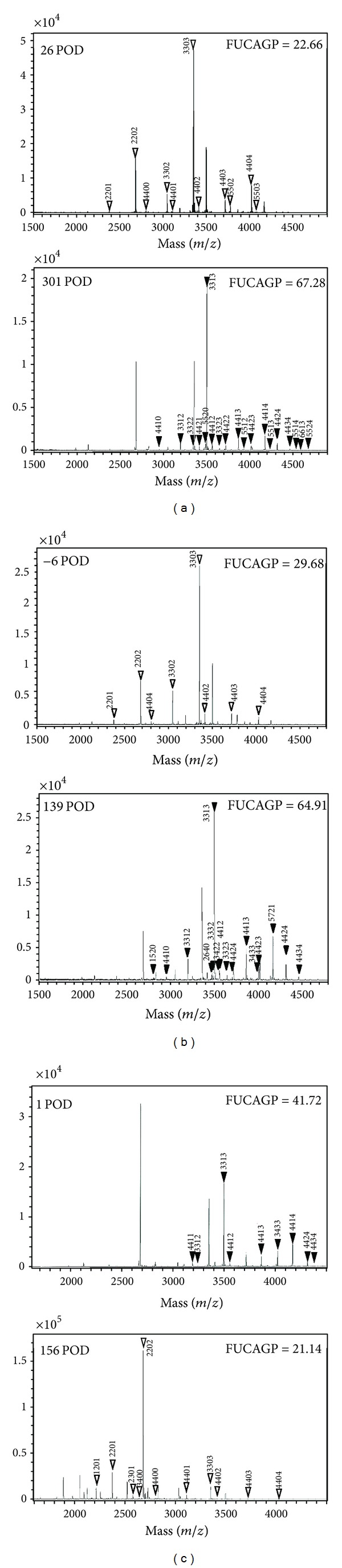
Mass spectra of AGP glycans isolated from followed up patients with advanced cancer. Patients with a poor prognosis, stage IV colon cancer with recurrence (a), rectal cancer with recurrence (b), and stage IIIA lung cancer (c). Purified AGP was prepared and labeled from 500 *μ*L of plasma samples and an each aliquot (ca. one-fiftieth) was used for MS analysis. White and black triangles indicate defucosylated and fucosylated tri- and tetra-antennary glycans assigned, respectively. POD, postoperative days.

Previously, follow-up studies of AGP glycans were undertaken in cancer patients over a long period after surgery, and their glycoforms were determined periodically, while their progress was monitored. Although demographic factors and plasma AGP level were not related to prognosis, based on fucosylation and branching indices, an individual patient's chance of survival could be predicted with the lowest misclassification rate (0.0666, *P* < 0.0001) [3]. While certain AGP glycoform could be a good prognostic marker for malignant disease, patients whose AGP glycoforms were highly fucosylated with branched glycans had a poor prognosis, died due to disease recurrence. Therefore, it was of particular interest to identify glycan structures having clinical relevance in the management of cancer patients. AGP glycans from a patient with stage IV colon cancer who had recurrence at 31 postoperative days (POD) and died were analyzed at 26 and 301 POD. The level of FUCAGP increased by more than threefold between 26 and 301 POD, reflecting levels of glycans, namely, FD numbers 3313, 4422, 4413, 4423, 4414, and 4424, together with relatively decreased expressions of 3302, 3303, 4403, and 4404 glycans (Figure 4(a)). Changes in glycoforms in this patient were associated with the degree of branching and the extent of fucosylation over the cut-off values at 301 POD [3]. Similarly, mass spectrometric analyses of AGP glycans from a patient with rectal cancer who had recurrence at 90 POD were also done both at 6 days before operation and at 139 POD. FUCAGP levels increased by more than twofold in line with an increase in the expressions of fucosylated tri-antennary glycans (FD numbers 3313, 3312, 3332) and fucosylated tetra-antennary glycans (4424, 4413, 4424 and 4434), along with decreases of 3302, 3303, 4403, or 4404 glycans (Figure 4(b)). In contrast, AGP glycans from a patient with stage IIIA lung cancer but with a good prognosis changed differently and fucosylated glycans such as 3313, 4414, 3433, 4413, 4424, 4411, and 4412 decreased markedly between 1 and 156 POD together with a fall of FUCAGP level (Figure 4(c)). In line with these observations, relative abundance of FUCAGP in patients who had AGP with either low degrees of branching or low level of fucosylation for a long period after operation was observed to be as low as those in healthy control. Therefore, all these mass spectrometric analyses in following up cancer patients showed that the primary structures of AGP glycans are in agreement with results from AGP glycoforms and that the FUCAGP index could be a postoperative marker for diagnosis and assessment of cancer progression. The newly developed AGPAS could be valuable software for a rapid determination of AGP glycan structures following mass spectrometric analyses. A detailed analysis of AGP glycans is now in progress with large numbers of plasma samples from cancer patients under various medications. 

## 4. Conclusions 

The functions of plasma AGP and its potential physiological significance as an acute phase protein have generated significant interest. However, most studies have focused on its highly glycosylated N-glycan structures along with recent developments of glycomics as well as approaches from proteomics [2, 17, 19, 22–29]. The detailed structures which were prepared from both healthy controls and patients with various diseases have been incompletely reported [14, 30–34]. Previously, we demonstrated that glycoforms of plasma AGP from cancer patients changed depending on the patients' clinical status and that any patient whose glycans contained highly fucosylated branched structures for long periods of time after operation showed a poor prognosis. In contrast, it was found that patients who had AGP glycoforms without such changes showed a good prognosis regardless of their clinical stages [3]. The present study demonstrated that assessment of primary structures of AGP glycans by following mass spectrometric analysis could be established with the aid of our newly developed software, AGPAS, which factors a database consisting of 607 N-glycans that were theoretically expected to be present in glycans of AGP. At the same time, a reliable purification method for plasma AGP by sequential use of ion-exchange cartridges was developed and followed by specifically labeling cleaved N-glycans from purified AGP. Therefore, relative abundance of all the glycans as well as bi-, tri-, and tetra-antennary glycans was simultaneously determined with their related sugar residues. It was clearly demonstrated that relative abundance of fucosylated AGP was significantly elevated in cancer patients and that mono- and difucosylated tri-antennary and monofucosylated tetra-antennary glycans were predominantly present in cancer patients. Furthermore, increased relative abundance of fucosylated AGP was found specifically in patients with a poor prognosis, consistent with not only our previous analyses of AGP glycoforms in large numbers of cancer patients but also follow-up studies of glycoforms in the same patients. The methods applied in this study seemed to be appropriate for processing large numbers of plasma samples to determine a biomarker in AGP glycans. In this endeavor, the operation software, AGPAS, should be valuable for screening plasma samples to identify biomarkers of cancer prognosis or progression based on AGP glycans with fucosylated structures. 

## Figures and Tables

**Table 1:** Relative abundances of N-glycans with bi-, tri-, and tetra-antennary chains and their fucosylated ones in plasma AGP from healthy controls and colorectal cancer patients.

			Relative abundance (%)^#^		
Glycan structure	Numbers of		Normal	Cancer	*P* value
	[GlcNAc]	[Fuc]	(*n* = 35)	(*n* = 67)	
Bi-antennary	2	0	36.12 ± 15.52	42.07 ± 14.03	n.s.

	3	0–2	47.10 ± 12.69	42.54 ± 13.03	n.s.
Tri-antennary	3	0	37.44 ± 12.57	25.84 ± 9.76	<0.001
	3	1	8.26 ± 3.63	14.60 ± 5.42	<0.001
	3	2	1.40 ± 1.05	2.10 ± 1.06	<0.001
**Fuc-tri-antennary**	3	1-2	**9.66 ± 3.02 **	**16.69 **±** 5.42**	**<0.001**

	4	0–3	15.03 ± 7.69	13.15 ± 4.08	n.s.
	4	0	7.95 ± 4.15	5.41 ± 2.19	<0.001
Tetra-antennary	4	1	1.42 ± 1.16	2.32 ± 1.30	<0.001
	4	2	0.57 ± 0.50	0.66 ± 0.57	n.s.
	4	3	0.29 ± 0.28	0.34 ± 0.36	n.s.
**Fuc-tetra-antennary**	4	1–3	**2.28 ± 1.65 **	**3.32 **±** 1.82**	**<0.01 **
	5–7	0–6	1.63 ± 1.24	1.58 ± 0.91	n.s.

Tri- + tetra-antennary	3–7	0	62.12 ± 17.09	55.69 ± 14.88	n.s.
Fuc-(tri- + tetra-antennary)*	3–7	1–6	**18.68 **±** 6.33 **	**23.02 **±** 5.7**	**<0.001 **

^#^Calculated based on the hit area to the total one. *FUCAGP: fucosylated (tri + tetra)-antennary glycans/total glycans × 100.
